# Does the Personality of Patients with Parkinson's Disease Affect the Decision to Perform Deep Brain Stimulation Surgery? A Cross-Sectional Study in a Chinese Cohort

**DOI:** 10.1155/2021/6639255

**Published:** 2021-01-26

**Authors:** Wei Lin, Dan Wang, Likun Yang, Jie Zhu, Jingjie Ge, Chuantao Zuo, Yuhai Wang

**Affiliations:** ^1^Department of Neurosurgery, Joint Logistics Support Unit No. 904 Hospital, School of Medicine, Anhui Medical University, Wuxi, China; ^2^PET Center, Huashan Hospital, Fudan University, Shanghai, China

## Abstract

We investigated whether the personality of patients with Parkinson's disease (PD) before subthalamic brain stimulation differed from patients receiving drug treatments and whether the personality of patients affected surgical decisions. We recruited 38 patients with advanced PD scheduled for deep brain stimulation (DBS), 40 patients with PD receiving the very best medical treatment, and 51 healthy control subjects. All participants were evaluated by the Minnesota multiphasic personality inventory-1 (MMPI-1). PD patients who were candidates for DBS did not exhibit any significant differences in personality when compared with PD patients who were treated with drugs. Compared with healthy controls, patients with PD had remarkably higher MMPI-1 scores for spiritual quality, neuroticism, and introversion, but significantly lower scores for socialization. In addition, patients with PD were more submissive, more dependent on others, and less active in social activities. Our data indicated that the main deciding factor relating to whether to undergo DBS was the disease itself and not the pathological personality. However, neurotic and psychotic symptoms accompanying PD may influence the effect of DBS. We found that greater benefit is obtained by surgical or medical interventions if abnormal neurotic characteristics are considered early in the course of PD.

## 1. Introduction

Parkinson's disease (PD) is the second most common degenerative disorder and is characterized by both motor and nonmotor symptoms. PD also has an extensive negative effect on the mental health of patients and presents with a wide range of psychiatric disorders involving depressive symptoms and general anxiety disorders. Rigidity, introversion, and cautiousness have all been proposed as typical personality characteristics of patients with PD [1].

Previous cross-sectional studies have compared the personalities of patients with PD to those of healthy subjects (HS) or patients with other neurological diseases such as Alzheimer's disease and suggested that PD patients are more introverted, apprehensive, tense, driven, and cautious. These data suggested the existence of “Parkinson's disease-specific personality” [2, 3]. Personality traits are known to affect perceptions of the overall impact of this disease on function, as well as general well-being (Lahey, 2009). Some studies of PD reported that there was no correlation that links personality traits to clinical features, such as disease duration and Hoehn and Yahr (H&Y) stage. However, other studies have presented contradictory findings [4–6].

Previous studies have suggested that certain personality qualities are typical of PD and may be linked to neurotransmitter alterations caused by disease progression or treatments. It is speculated that these personality traits are early manifestations of neurochemical changes during PD [7, 8].

The Minnesota multiphasic personality inventory (MMPI) (Hathaway 1940) is the most widely studied personality test and is commonly used to examine personality traits, as well as psychopathology. However, only specially trained psychologists can administer this personality test. Some previous studies have used the MMPI-1 to investigate personality characteristics in PD patients [9].

Since its introduction in the early 1990s, deep brain stimulation (DBS) is widely regarded as the second milestone in the treatment of PD after dopamine replacement therapy. However, the postoperative effects of DBS are known to depend on a variety of different factors, including disease-specific pathopsychological symptoms [10, 11].

In this study, we investigated the personality of PD patients at our hospital prior to DBS surgery (PD-DBS) or the best form of medical treatment (PD-MED). This study sought to determine if there were personality differences between these two groups of PD patients and the personality characteristics of healthy subjects at our center.

## 2. Materials and Methods

### 2.1. Subjects

PD patients were recruited into the study a week before DSB surgery (PD-DBS; 38 patients) or treatment with drugs (PD-MED; 40 patients). Fifty-one healthy participants were also recruited as healthy controls (HC). We recorded a range of demographic data, including age, education level, gender, medical history, and levodopa and dopamine agonist usage. Further neuropsychological examinations were performed on every subject.

Patients were included if they had received a clinical diagnosis of idiopathic PD based on the British Parkinson's Disease Society Brain Bank Criteria for at least 5 years [12]. Patients were excluded if they had other known neurodegenerative and/or psychiatric disorders.

### 2.2. Personality Assessment

All PD subjects were asked to take anti-Parkinsonian medication prior to evaluation.

#### 2.2.1. MMPI Assessment

Each patient was given a psychometric assessment using the Chinese version of the MMPI [13], which involves 566 statements that are answered as “true” or “false.” The results were scored in a standard profile consisting of 3 “validity scales” and 10 “personality scales.” The validity scales involved lie (L), fake (F), K correction, and defensive responses (K). Personality scales involved states of hypochondriasis (Hs), schizophrenia (Sc), depression (D), hysteria (Hy), masculinity-femininity (Mf), paranoia (Pa), psychasthenia (Pt), psychopathic deviate (Pd), mania (Ma), and social introversion (Si). Six clinical factors were also considered: P (including positive loads of F, PA, Pt, Ma, and SC and negative loads of L and K), N (including Hs, Hy, and D), I (including Pt, D, and Si), F (Pa), M (Mf), and A (including high negative loads of Pd and a medium negative load of Pa). This approach was used as described previously (James N. Butcher, 1976).

#### 2.2.2. QUIP PD-Short Assessment

All subjects were tasked to complete a questionnaire relating to impulsive comprehensive disorders in Parkinson's disease (QUIP PD-short). This is a comprehensive screening test that evaluates a wide range of impulsive compulsive behaviors [14]. Scoring above a predetermined threshold indicates that the patient answered the test with bias, thus invalidating the personality scale results. The results of the MMPI test are presented as normalized *t*-scores. Scale scores indicate psychological dysfunction when the *t*-value was >60. Absolute scores are presented as means of the normalized *t*-scores.

### 2.3. Statistical Methods

All clinical parameters were indicated as the mean ± standard deviation (SD) or as percentages in the case of categorical variables. Dr. Zhang and his team considered 60 points as the demarcation point. Sensitivity, specificity, and interpreter reliability were calculated by comparing normal subjects versus those with mental disorders. Thus, the cutoff threshold was set at 60.

All patients, as well as healthy controls, were compared using one-way analysis of variance (ANOVA) for continuous parameters. Chi-squared tests were used to compare categorical parameters, and *p* < 0.05 was regarded as being statistically significant.

### 2.4. Ethical Considerations

This study was approved by the local ethics committee. All procedures adhered to the ethical guidelines of the responsible committee on human experimentation (institutional and national) and the Helsinki Declaration of 1975 (revised in 2000).

## 3. Results

In total, 129 subjects were recruited into the study: 51 in the healthy control group, 38 in the PD-DBS group, and 40 in the PD-MED group (Table 1). The basic characteristics of the 38 PD-DBS patients were as follows: mean age = 62.76 ± 9.31 years, mean H&Y score = 3.52 ± 0.80, mean disease duration = 11.53 ± 4.65, and mean Unified Parkinson's Disease Rating Scale (UPDRS) part III score = 45.63 ± 12.61. The basic characteristics of the 40 PD-MED were as follows: mean age = 67.43 ± 10.03 years, mean H&Y score = 2.60 ± 1.24, mean disease duration = 7.97 ± 6.36 years, and mean UPDRS score = 35.97 ± 18.04. The mean age of the 51 participants in the HS group was 57.49 ± 6.74 years. There were no statistically significant differences between the two groups with regard to age, gender, total levodopa equivalent dosage (total LEDD), or the use of levodopa and DA agonist LED (*p* ≥ 0.05). Even more, no statistically significant differences were detected between the two groups with regard to H&Y stage, disease duration, and UPDRS III scores (*p* ≥ 0.05), thus indicating that the condition of the patients in the PD-DBS group was similar to PD patients with the best medical treatment.

**Figure 1:**
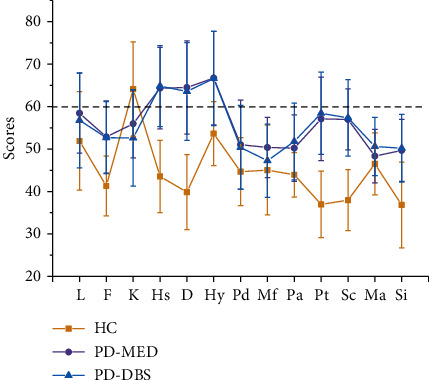
Scores of validity scales and personality scales in three groups of subjects. Note: HC: healthy control; PD-MED: drug-treated PD patients; PD-DBS: STN-DSB surgery; MMPI-1: Minnesota multiphasic personality inventory-1; L: lie; F: fake; K: K correction and defensive responses; Hs: hypochondriasis; D: depression; Hy: hysteria; Pd: psychopathic deviate; Mf: masculinity/femininity; Pa: paranoia; Pt: psychasthenia; Sc: schizophrenia; Ma: hypomania; Si: social introversion.

**Figure 2:**
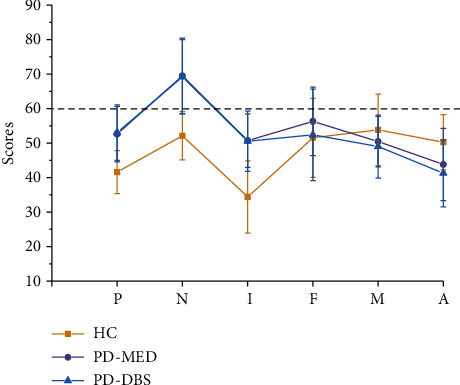
Scores of clinical factors in three groups of subjects. Note: P: including positive loads of F, PA, Pt, Ma, and SC and negative loads of L; N: including Hs, Hy, and D; I: including Pt, D, Si, F, Pa, M, and Mf; A: including high negative loads of Pd and medium negative load of Pa.

We found no significant differences between the two groups with regard to the QUIP PD-short neuropsychological evaluation (*p* > 0.05, Table 2). However, one-way ANOVA revealed significant differences in all 13 subscales when compared between the three groups (PD-MED, PD-DBS, and HC) (Figures 1 and 2).

Significant differences were detected between the groups when considering the three corrected scales (*p* ≤ 0.01). Analyses of 10 clinical scales revealed differences between the groups (*p* ≤ 0.01) and significant differences in the Mf scale (*p* ≤ 0.01) and Ma scale (*p* = 0.09). Moreover, the mean scores for the PD-DBS and PD-MED were >60 for Hy, D, and Hs. Using the N factor scale, the mean scores for the two PD groups were >65 and were both significantly higher than that of the HC group. However, post hoc analysis failed to identify any significant differences between the PD groups.

## 4. Discussion

To the best of our knowledge, this is the first study to investigate the personalities of PD patients prior to DBS or medical treatment. No significant differences were detected between the PD-DBS and PD-MED groups with regard to the dimensions of temperament, and the two groups exhibited similar psychotic characteristics. These data indicated that patients undergoing surgery do not have special personality traits; rather, they are part of the wider PD population.

Statistically significant differences were reported between the PD-DBS and PD-MED patients with regard to the H&Y stage, disease duration, and UPDRS III scores. These data indicated that PD was more severe in the PD-DBS group and that these patients required surgical intervention because medication was not sufficiently effective.

No significant differences were detected between the PD-DBS and PD-MED groups with regard to LEDD and DA drug usage, thus ruling out any influence that may have been created by these drugs. Analysis of QUIP scales further indicated that Compulsive Disorder (OCD) does not affect the decision to operate. After excluding impulsive factors, we investigated whether personality factors had an effect on surgical decisions within our patient population.

It has been reported that patients who develop PD often show a typical premorbid personality profile that is characterized by industriousness, inflexibility, cautiousness, and low impulsivity; these persist after the disease begins to affect the motor system. However, other studies show that in addition to the innate factors that may affect personality, personality changes that develop with PD gradually become obsessive, dependent, affectively inconstant, passive, industrious, worried, and sick body perception, when compared with healthy controls [15, 16].

Although it is difficult to differentiate between premorbid personality features from those that develop after the appearance of the disease's typical manifestations, this should not prevent further attempts to reexamine the premorbid personality using newer hostility, as well as personality rating scales. A previous study investigated 6,822 participants over 40 years and used MMPI scales to monitor extraversion (sensation seeking, hypomania, and positive emotionality), introversion (social introversion and constraint), and neuroticism (psychasthenia, pessimistic personality, and depression) and found that only neuroticism was significantly correlated with the development of PD later in life [17]. Our results are consistent with these previous findings; the neuroticism score of our PD patients (>65) was significantly higher than that of the HC group, thus indicating a characteristic personality that is associated with PD.

Neuroticism derived from the Hs, D, and Hy scales showed considerable correlation with the subsequent development of PD. This relationship was primarily due to the anxious personality scale (psychasthenia). The correlation between the pessimistic personality trait and the absence of depression was poor. The neuroticism personality trait refers to negative emotions to threat, frustration, or loss and is typified by anxiety, moodiness, worry, envy, and holding grudges [18]. Neuroticism has been demonstrated to elevate the risk of depression in PD and can potentially aggravate the disease. Collectively, these observations imply that neuroticism is a hallmark personality of PD; this is consistent with our observations of PD-MED and PD-DBS patients. A mean neuroticism factor score that is higher than the HC group indicates emotional instability, disease progression, or poor disease control, thus suggesting that high levels of neuroticism may predispose patients to a reduced quality of life or poorer disease control [19]. Thus, we need to place greater emphasis on neuroticism, especially after DBS. A profound understanding of the role of neuroticism may help to improve the treatment of PD patients with high levels of neuroticism.

The D scale is related to psychopathic features and neurotic disorders. Depression is a clinical symptom of a variety of disorders, including bad moods, schizophrenia, bipolar depression, OCD, and anxiety disorder. Some studies have found that characteristic personalities, such as neuroticism, OCD, and anxiety disorder, are related to defects in the distribution of dopamine. However, other studies, involving neuroanatomical and functional studies, have indicated that it is not easy to conclude that dopamine distribution is the only cause [4, 20, 21]. Some studies suggest that DBS in PD results in personality changes, including an augmented impulsive profile, despite reduced levels of L-dopa and DA, thus supporting the idea of a primary behavioral effect caused by DBS [22, 23]. A recent study reported self-perceived personality changes in 6 out of 27 (22%) STN-DBS patients and identified that preoperative hypomania was the most remarkable predictor for personality change. Nevertheless, standard measurement scales did not sufficiently reflect personality or mood changes that were perceived subjectively by patients [24].

Chronic diseases can exert a notable long-term influence on personality, profoundly impacting the outcome of DBS. Relative to healthy controls, we found that our PD patients exhibited significant differences in three dimensions (spirit, neuroticism, and introversion), a significantly lower socialization dimension, greater levels of submissiveness, greater dependence on others, and less engagement in social activities. The MMPI characteristics of PD patients taking medications were consistent with those of PD patients prior to STN-DBS surgery. PD patients also exhibited some personality changes prior to surgery. Thus, it is very important to consider the mental state of PD patients prior to surgery.

Future research should be aimed at overcoming these limitations such as the number of the patients and longitudinal personality changes and thus validate the occurrence of changes in personality traits after DBS, to better determine whether specific personality traits can predict the progression of the disease or the effects of interventions such as DBS.

## 5. Conclusion

Chronic diseases can exert significant long-term influences on personality. These personality factors, particularly neuroticism, although are not factors in deciding whether to undergo operation, may have a significant effect on the postoperative effects of DBS.

## Figures and Tables

**Table 1:** Demographics and clinical feathers in all subjects.

	PD			HC (51)	*p* value
	PD-DBS (38)	PD-MED (40)	*p* value		
Age (years)	62.76 ± 9.31	67.43 ± 10.03	0.057	57.49 ± 6.74	0.067
Duration (years)	11.53 ± 4.65	7.97 ± 6.36	0.074	3.82 ± 3.71	—
H&Y stage	3.52 ± 0.80	2.60 ± 1.24	0.198	2.18 ± 1.33	—
Sex (F/M), no.	17 : 21	14 : 26	0.380	31 : 20	0.052
Total LEDD (mg)	795.0 ± 288.0	608.3 ± 150.6	0.199		
Levodopa (mg/d)	610.0 ± 176.1	454.2 ± 150.3	0.154	—	—
DA agonist LED (mg/d)	100.8 ± 83.7	125.0 ± 38.7	0.528		—
UPDRS III	45.63 ± 12.61	35.97 ± 18.04	0.0735		

Note: data are given as the mean ± standard deviation. UPDRS III: Unified Parkinson's Disease Rating Scale, part III. Note: DA agonist‐LED (DA‐LED, mg/d) = piribedil (mg/d) × 1 + pramipexole (mg/d) × 100. Total LED (TLED, mg/d) = regular levodopa dose (mg/d) × 1 + levodopa CR dose (mg/d) × 0.75 + DA‐LED, and plus [regular levodopa dose (mg/d) + CR levodopa dose (mg/d) × 0.75] × 0.33 if taking COMT-I.

**Table 2:** Comparison between patients with and without ICD behaviors.

Variable	PD-DBS (38)	PD-MED (40)	*p* value
QUIP disorders			
Any 1 or more disorders	2	3	0.833
Gambling	1	1	1.0
Sex	0	1	—
Buying	1	1	1.0
Eating	0	0	—
Hobbyism	0	0	—
Punding	0	0	—

## Data Availability

All the clinical data used to support the findings of this study may be released upon application to the data access manager, who can be contacted at +8613812512187.
