# Corrigendum to “Does the Personality of Patients with Parkinson's Disease Affect the Decision to Perform Deep Brain Stimulation Surgery? A Cross-Sectional Study in a Chinese Cohort”

**DOI:** 10.1155/2021/9781803

**Published:** 2021-05-24

**Authors:** Wei Lin, Dan Wang, Likun Yang, Jie Zhu, Jingjie Ge, Chuantao Zuo, Yuhai Wang

**Affiliations:** ^1^Department of Neurosurgery, Joint Logistics Support Unit No. 904 Hospital, School of Medicine, Anhui Medical University, Wuxi, China; ^2^PET Center, Huashan Hospital, Fudan University, Shanghai, China

In the article titled “Does the Personality of Patients with Parkinson's Disease Affect the Decision to Perform Deep Brain Stimulation Surgery? A Cross-Sectional Study in a Chinese Cohort” [1], the authors identified an error in the Discussion that was introduced during the preparation of the manuscript. Statistical differences were not reported between PD-DBS and PD-MED, and the article should be corrected as follows:

“Statistically significant differences were reported between the PD-DBS and PD-MED patients with regard to the H&Y stage, disease duration, and UPDRS III scores. These data indicated that PD was more severe in the PD-DBS group and that these patients required surgical intervention because medication was not sufficiently effective.”

Should be corrected to

“Statistically significant differences were not reported between the PD-DBS and PD-MED patients with regard to H&Y stage, disease duration, and UPDRS III scores.”
